# Duration and urgency of transfer in births planned at home and in freestanding midwifery units in England: secondary analysis of the Birthplace national prospective cohort study

**DOI:** 10.1186/1471-2393-13-224

**Published:** 2013-12-05

**Authors:** Rachel E Rowe, John Townend, Peter Brocklehurst, Marian Knight, Alison Macfarlane, Christine McCourt, Mary Newburn, Maggie Redshaw, Jane Sandall, Louise Silverton, Jennifer Hollowell

**Affiliations:** 1 National Perinatal Epidemiology Unit, University of Oxford, Old Road Campus, Oxford OX3 7LF, UK; 2 Institute for Women’s Health, University College London, London, UK; 3 Department of Midwifery and Child Health, City University London, London, UK; 4 The National Childbirth Trust, London, UK; 5 Division of Women’s Health, King’s College London, London, UK; 6 The Royal College of Midwives, London, UK

**Keywords:** Birthing centres, Home birth, Midwifery, Patient transfer

## Abstract

**Background:**

In England, there is a policy of offering healthy women with straightforward pregnancies a choice of birth setting. Options may include home or a freestanding midwifery unit (FMU). Transfer rates from these settings are around 20%, and higher for nulliparous women. The duration of transfer is of interest because of the potential for delay in access to specialist care and is also of concern to women. We aimed to estimate the duration of transfer in births planned at home and in FMUs and explore the effects of distance and urgency on duration.

**Methods:**

This was a secondary analysis of data collected in a national prospective cohort study including 27,842 ‘low risk’ women with singleton, term, ‘booked’ pregnancies, planning birth in FMUs or at home in England from April 2008 to April 2010. We described transfer duration using the median and interquartile range, for all transfers and those for reasons defined as potentially urgent or non-urgent, and used cumulative distribution curves to compare transfer duration by urgency. We explored the effect of distance for transfers from FMUs and described outcomes in women giving birth within 60 minutes of transfer.

**Results:**

The median overall transfer time, from decision to transfer to first OU assessment, was shorter in transfers from home compared with transfers from FMUs (49 vs 60 minutes; p < 0.001). The median duration of transfers before birth for potentially urgent reasons (home 42 minutes, FMU 50 minutes) was 8–10 minutes shorter compared with transfers for non-urgent reasons. In transfers for potentially urgent reasons, the median overall transfer time from FMUs within 20 km of an OU was 47 minutes, increasing to 55 minutes from FMUs 20-40 km away and 61 minutes in more remote FMUs. In women who gave birth within 60 minutes after transfer, adverse neonatal outcomes occurred in 1-2% of transfers.

**Conclusions:**

Transfers from home or FMU commonly take up to 60 minutes from decision to transfer, to first assessment in an OU, even for transfers for potentially urgent reasons. Most transfers are not urgent and emergencies and adverse outcomes are uncommon, but urgent transfer is more likely for nulliparous women.

## Background

In England, there is a policy of offering healthy women with low risk pregnancies a choice of birth setting. Choices may include an obstetric unit (OU), an alongside midwifery unit (AMU) situated on the same site as an OU, a freestanding midwifery unit (FMU) situated on a site without an OU, or at home. For ‘low risk’ women, planned birth in a midwifery unit or at home is associated with benefits for the mother in terms of fewer interventions [1-7]. Outcomes for babies of women who plan birth outside an OU are comparable with those for babies of women who plan birth in an obstetric unit, with the exception of babies of nulliparous women planning birth at home, for whom adverse perinatal outcomes are more common [1,2].

National clinical guidelines for intrapartum care in England recommend referral for obstetric advice, with transfer to an obstetric unit where appropriate, when certain clinical complications occur, including diagnosed delay in the first or second stage of labour, abnormal fetal heart rate, “significant” meconium staining, fresh bleeding, maternal pyrexia, maternal hypertension, retained placenta and suspected postpartum haemorrhage [5]. In planned home and FMU births, obstetric, anaesthetic and neonatal care are only available if the woman is transferred, usually by car or ambulance, to an obstetric unit. Overall transfer rates from these settings are around 20%, but rates for nulliparous women are substantially higher (36% in FMUs and 45% in planned home births) [1,2].

Transfer from planned home births and FMUs raises concerns about safety, in part because of the potential for delay [8-12]. In the UK, it has also been suggested that high transfer rates from FMUs pose “logistical problems” and that more AMUs should be developed [13,14]. While there may be a perception that AMUs are safer than FMUs, presumably because of the speed with which obstetric and neonatal care are potentially available if needed, evidence from a recently completed study of AMUs indicates that transfer from AMUs may not be straightforward, with delays occurring because of staffing and resource constraints and intra-professional tensions [15]. Analysis of the Birthplace primary outcome (a composite measure of adverse perinatal outcomes) also found similar event rates in the two midwifery unit settings [1,2]. Transfer is also an issue which can influence women’s decision-making about place of birth [16-18]. Some women describe choosing birth in an AMU to avoid the possibility of transfer by car or ambulance [18]. Those planning birth at home or in an FMU want information about transfer, may be concerned or ill-informed about journey time and may find longer journeys more difficult [18].

This study aimed to estimate the overall duration of transfer from planned births at home and in FMUs, to explore and describe the association between urgency and transfer duration from both settings and the association between distance to the nearest OU and transfer duration from FMUs.

## Methods

### Study design

This was a secondary analysis of data collected in the Birthplace prospective cohort study, which aimed to compare perinatal and maternal outcomes by planned place of birth.

### Setting and participants

The cohort study methods are described in full elsewhere [1,2]. Data were collected on 79,774 births between 1st April 2008 and 30th April 2010. Of these 11,666 were planned in 53 FMUs, 17,582 planned in 43 AMUs and 18,269 planned at home in 142 NHS trusts in England. Births to all women who were attended by an NHS midwife during labour in their planned place of birth, for any amount of time, were eligible for inclusion. Women who had an elective caesarean section or caesarean section before labour, who presented in preterm labour (<37 weeks’ gestation), had a multiple pregnancy, or who received no antenatal care were excluded, as were women who had a stillbirth before the start of care in labour.

In the cohort, women were classified as ‘low risk’ if before the start of labour they were not known to have any of the medical or obstetric risk factors listed in national guidelines on intrapartum care [5] as “indicating increased risk suggesting planned birth in an obstetric unit”.

The study population for the analyses reported here was ‘low risk’ eligible women with a ‘term’ pregnancy (37-42^+0^ weeks’ gestation) who planned to give birth in an FMU or at home.

### Data

Data relating to labour and birth were collected by midwives attending women in labour [1]. When a woman was transferred, either during labour or after birth, data were collected about the primary reason for transfer and the date and time of the decision to transfer, the start of transfer and when the woman was first seen by a midwife and/or an obstetrician after arrival at the OU.

The primary outcome for these analyses was the duration of transfer. The timing and duration of transfer was described using five measures, broadly based on those used in an audit of community maternity units in Scotland [19].

• *Time to decision:* the time from the start of care in labour to the decision to transfer;

• *Arranging transfer:* the time from the decision to transfer to the start of transfer (when the woman left her planned place of birth);

• *Departure to first OU assessment:* the time from when the woman left her planned place of birth to when she was first seen by a midwife or obstetrician in the receiving OU;

• *Overall transfer time:* the time from the decision to transfer to when the woman was first seen by a midwife or obstetrician in the receiving OU;

• *After transfer:* the time from when the woman was first seen by a midwife or obstetrician in the receiving OU to when she gave birth (for transfers before birth only).

For analyses relating to transfer duration, records were checked to ensure that the recorded times for the transfer process followed a logical sequence. Where they did not, and could not be corrected, records were excluded from analyses of transfer duration (205 (5.9%) from the home birth group and 112 (4.6%) from the FMU group).

Although data on the reasons for transfer were collected, there were no explicit data on the urgency of transfer. In order to explore the association between urgency and the duration of transfer, the recorded primary reasons for transfer were grouped according to their likely urgency, based on clinical judgement. We considered transfers before and after birth separately. Transfers before birth where the recorded primary reason was antepartum haemorrhage, failure to progress in the 2nd stage and fetal distress in the 1st or 2nd stage were defined as transfers for potentially urgent reasons and compared with transfers before birth where the recorded primary reason was failure to progress in the 1st stage or epidural request, defined as transfers for non-urgent reasons. Transfers after birth for postpartum haemorrhage were considered as a separate potentially urgent group. The study records relating to all transfers for potentially urgent reasons where the overall transfer time was greater than 90 minutes were manually reviewed, together with a sample of similar records of transfers for non-urgent reasons, to establish whether reasons for delay could be ascertained or inferred or obvious errors detected. While some longer transfer times seemed implausible, given available data it was not possible to verify or discount these. Given the small number of these cases and the non-parametric methods used it is not likely that these outliers will have had a measureable effect on the overall conclusions so they were retained in the dataset.

We used data about interventions (caesarean and instrumental delivery) and perinatal outcomes (Apgar score less than 7 at 5 minutes and a composite of intrapartum stillbirth, neonatal admission or early neonatal death) in births occurring within 60 minutes of first assessment in the OU after transfer to validate the definitions of urgency used and to estimate the proportion of transfers and planned births in each setting which might be considered as being in need of urgent care after transfer. In women transferred after birth for postpartum haemorrhage we explored the proportion who received a blood transfusion. In order to provide a comparison with transfers from the other settings, these analyses were also carried out for transfers from AMUs, where there is no car or ambulance journey involved and so potentially shorter transfer times. The results of these analyses are presented in Additional file 1: Table S1 and Additional file 2: Table S2.

Data on distance for transfers from home were not available because the regulatory approvals for the Birthplace cohort study did not allow for collection of ‘identifiable’ data such as postcodes. The distance from each FMU to the nearest OU in the same NHS trust was calculated using Google maps [20] based on postcodes. The number of births planned in each FMU per year was estimated using the number of planned births in the unit reported during each month of the Birthplace study period.

### Statistical methods

Overall transfer rates, reasons for transfer and the timing and urgency of transfer were tabulated by parity for each setting as a proportion of all planned births. The median and interquartile range (IQR) were calculated for each measure of transfer duration and medians were compared between specific groups using Wilcoxon’s rank sum test. Cumulative distribution curves were plotted showing overall transfer time (from decision to transfer to first OU assessment) against the percentage of before birth transfers by urgency for each planned place of birth. Median overall transfer times for each FMU were plotted against distance to the nearest OU for transfers before birth for potentially urgent reasons. Correlations between transfer durations and distance were assessed using Spearman’s rank correlation coefficient (r_s_) due to the non-normal distributions of the data.

As in previous analyses of the Birthplace cohort, we used probability weights to adjust for the varying duration of participation of individual units and trusts and robust variance estimation, where appropriate, to allow for the ‘clustering’ of women within trusts/units. Unweighted frequencies and percentages were used to describe characteristics of the sample; weighted percentages and medians were used elsewhere.

All analyses were conducted using Stata SE version 11.2 [21].

### Ethical approval

Approval for the Birthplace prospective cohort study was obtained from the Berkshire Research Ethics Committee (MREC ref 07/H0505/151) and did not require consent to be sought from participants. No further ethics approval was required for the analyses reported here.

## Results

Overall there were 27,842 eligible ‘low risk’ women with term pregnancies in the cohort, 16,632 planning birth at home and 11,210 in an FMU; similar proportions of women were transferred from the two settings (home: 20.8%, 95% CI 20.2-21.4; FMU: 21.8%, 95% CI 21.0-22.6). Reflecting the underlying population of women planning birth in these settings, most transferred women were white, had a fluent understanding of English and were married or living with a partner (Table 1). More women planning birth at home, and more women transferred from planned home births, were aged over 30 and were having a second or subsequent baby compared with women planning birth in, and transferred from, FMUs.

**Table 1:** **Characteristics of women**^**1 **^**transferred and not transferred from home or a freestanding midwifery unit**

	**Not transferred**				**Transferred**			
	**Home**		**FMU**		**Home**		**FMU**	
	**N = 13175**		**N = 8766**		**N = 3457**		**N = 2444**	
	**n**	**%**	**n**	**%**	**n**	**%**	**n**	**%**
**Maternal age**								
Mean [SD]	31.1	[5.2]	28.8	[5.8]	30.9	5.1	28.5	5.7
Under 20	152	1.2	515	5.9	65	1.9	158	6.5
20-24	1346	10.2	1634	18.7	344	10.0	483	19.8
25-29	3400	25.9	2527	28.9	905	26.2	725	29.7
30-34	4483	34.1	2512	28.7	1290	37.3	712	29.2
35-39	3222	24.5	1360	15.5	738	21.4	318	13.0
≥40	542	4.1	207	2.4	114	3.3	45	1.8
Missing	30		11		1		3	
**Ethnic group**								
White	12461	94.7	8016	91.5	3280	95.0	2246	91.9
Asian	94	0.8	316	3.6	25	0.7	79	3.3
Black	197	1.5	112	1.2	39	1.2	29	1.2
Mixed	212	1.6	103	1.2	65	1.9	21	0.9
Other	194	1.5	215	2.5	44	1.3	68	2.8
Missing	17		4		4		1	
**Understanding of english**								
Fluent	13090	99.5	8503	97.2	3429	99.4	2353	96.5
Some or none	69	0.5	241	2.8	19	0.6	86	3.5
Missing	16		22		9		5	
**Marital/partner status**								
Married/Living together	12572	96.1	8102	93.5	3287	95.6	2277	93.9
Single/Unsupported	511	3.9	566	6.5	152	4.4	147	6.1
Missing	92		98		18		20	
**Body mass index (kg/m**^**2**^**)**								
Mean [SD]	24	3.7	24.1	3.8	24.1	3.7	23.9	3.5
not recorded	2533	19.3	1331	15.2	698	20.3	516	21.2
<18.5	261	2.0	185	2.1	56	1.6	47	1.9
18.5-24.9	6421	49.0	4393	50.2	1629	47.4	1180	48.4
25-29.9	2943	22.4	2088	23.8	799	23.3	549	22.5
30-35.0	954	7.3	758	8.7	253	7.4	147	6.0
Missing	63		11		22		5	
**IMD quintiles**								
1st Least deprived	2904	22.2	1931	22.1	739	21.5	552	22.6
2nd	2739	20.9	2005	22.9	697	20.3	565	23.2
3rd	2816	21.5	1768	20.2	789	23.0	520	21.3
4th	2617	20.0	1624	18.6	672	19.6	441	18.1
5th Most deprived	2008	15.3	1411	16.1	536	15.6	362	14.8
Missing	91		27		24		4	
**Previous pregnancies > =24 completed weeks**								
0 Nulliparous	2481	18.8	3284	37.5	2008	58.1	1868	76.7
1 previous	5587	42.4	3489	39.8	870	25.2	405	16.6
2 previous	3269	24.8	1385	15.8	361	10.4	118	4.8
3+ previous	1826	13.9	604	6.9	217	6.3	44	1.8
Missing	12		4		1		9	
**Gestation (completed weeks)**								
Mean [SD]	39.7	1.0	39.7	1.0	39.9	1.0	39.9	1.0
37	306	2.3	255	2.9	72	2.1	60	2.5
38	1293	9.8	795	9.1	275	8.0	183	7.5
39	3443	26.1	2196	25.1	646	18.7	473	19.4
40	5214	39.6	3421	39.0	1382	40.0	943	38.6
41 and 42 + 0	2919	22.2	2099	23.9	1082	31.3	785	32.1
Missing	0		0		0		0	

^1^‘Low risk’ women with term pregnancies.

### Transfer rates and reasons for transfer

In both settings nulliparous women were more likely to be transferred compared with women having a second or subsequent baby (home: 44.1% vs 11.6%; FMU: 34.5% vs 9.2%) (Table 2). The most common reason for transfer, in both settings and irrespective of parity, was failure to progress. In nulliparous women, 18% of those planning birth at home and 13% of those planning FMU birth were transferred for failure to progress in either the first or second stage. In multiparous women, failure to progress was the single most common reason for transfer, but almost half of all transfers took place after the birth for reasons such as repair of perineal trauma, retained placenta, postpartum haemorrhage and concerns about the baby. Overall, in both settings and irrespective of parity, most (60-70%) transfers for failure to progress were in the first stage of labour.

**Table 2:** **Primary reason for transfer, timing and urgency by planned place of birth and parity**^**1**^

	**Home**				**Freestanding midwifery unit**			
	**N = 16,619**				**N = 11,197**			
	**Nulliparous**		**Multiparous**		**Nulliparous**		**Multiparous**	
	**n**	**%**	**n**	**%**	**n**	**%**	**n**	**%**
**Women not transferred**	2481	55.9	10682	88.4	3284	65.5	5478	90.8
**Women transferred**	2008	44.1	1448	11.6	1868	34.5	567	9.2
**Primary reason for transfer**^**2**^								
Malposition	11	0.3	15	0.1	8	0.1	3	<0.1
Malpresentation	34	0.8	35	0.3	28	0.5	13	0.2
Failure to progress 1st stage	521	11.2	206	1.7	457	8.0	76	1.2
Fetal distress 1st stage	95	2.2	85	0.7	165	3.2	36	0.6
Meconium staining	246	5.4	178	1.4	247	4.5	53	0.8
Epidural request	131	2.8	44	0.4	139	2.4	23	0.3
Hypertension	41	0.9	32	0.2	48	1.0	16	0.2
Antepartum haemorrhage	34	0.8	26	0.2	32	0.6	14	0.2
Failure to progress 2nd stage	300	6.7	78	0.6	316	5.3	48	0.7
Fetal distress 2nd stage	30	0.6	11	0.1	29	0.5	6	0.1
Postpartum haemorrhage	53	1.2	88	0.7	37	0.7	53	0.9
Retained placenta	85	1.8	161	1.2	81	1.7	96	1.5
Repair of perineal trauma	203	4.4	180	1.4	144	2.9	37	0.6
Other before birth^3^	149	3.4	110	0.9	58	1.3	33	0.5
Other after birth, maternal reasons	9	0.2	18	0.1	9	0.1	11	0.2
Other after birth, neonatal reasons	42	0.9	141	1.1	33	0.6	32	0.6
Not known	24	0.6	40	0.4	37	1.0	17	0.5
**Timing of transfer**^**2**^								
During labour (before birth)	1563	34.2	764	6.0	1521	26.9	316	4.9
Immediately after birth	401	8.6	633	5.0	304	6.0	237	3.9
Not known	44	1.3	51	0.6	43	1.5	14	0.5
**Urgency of reason for transfer**^**2**^								
Potentially urgent (before birth)^4^	462	10.3	206	1.6	540	9.5	102	1.5
Non-urgent (before birth)^5^	640	13.6	244	2.0	589	10.3	98	1.5
Potentially urgent (after birth)^6^	53	1.2	88	0.7	37	0.7	53	0.9
Not classified^7^	853	19.1	910	7.3	702	14.0	314	5.3

^1^Including only women with known parity. Proportions are weighted to allow for different durations of data collection.

^2^As a proportion of all nulliparous/multiparous women planning birth in each setting.

^3^Including: prolonged rupture of membranes; failure to progress with no stage of labour specified; fetal distress with no stage specified or other concerns about the baby during labour; concerns about the mother during labour; pain relief other than epidural; maternal request, other than for epidural; non-medical reasons, including NHS resource issues such as staffing; clear breach of MU criteria or other factors that might indicate unsuitability for out of hospital birth.

^4^Transfers before birth for fetal distress (1st or 2nd stage or stage not specified), failure to progress in the 2nd stage or antepartum haemorrhage (excluding those where timing was not known or inconsistent).

^5^Transfers before birth for failure to progress in the 1st stage or epidural request (excluding those where timing was not known or inconsistent).

^6^Transfers after birth for postpartum haemorrhage.

^7^All other reasons for transfer (including not known) not classified as potentially urgent or non-urgent above.

### The transfer process: timing and duration

On average decisions to transfer were taken slightly sooner after the start of care in labour for women transferred from home compared with women transferred from an FMU (Table 3). This difference between settings was not apparent for potentially urgent transfers (before birth).

**Table 3:** **The timing and duration of transfer**^**1**^

	**Home**		**Freestanding midwifery unit**	
	**Median**	**IQR**	**Median**	**IQR**
**Time to decision to transfer**^**2 **^**(hours)**				
All transfers	4.7	(2.3, 7.7)	5.3	(2.9, 8.7)
Transfers during labour (before birth)	5.0	(2.3, 8.0)	5.4	(2.8, 8.8)
Potentially urgent^3^ transfers (before birth)	5.4	(2.8, 8.0)	5.2	(2.7, 8.2)
Non-urgent^4^ transfers (before birth)	6.6	(4.3, 9.5)	7.5	(4.8, 10.2)
Transfers after birth	4.0	(2.3, 6.0)	5.2	(3.2, 8.1)
**Arranging transfer**^**5 **^**(mins)**				
All transfers	20	(10, 30)	24	(15, 35)
Transfers during labour (before birth)	19	(10, 30)	20	(14, 32)
Potentially urgent transfers (before birth)	15	(10, 25)	20	(10, 30)
Non-urgent transfers (before birth)	20	(13, 30)	25	(15, 38)
Transfers after birth	25	(15, 40)	30	(15, 45)
Potentially urgent transfers (after birth)^6^	20	(14, 30)	25	(15,39)
**From departure to first OU assessment**^**7 **^**(mins)**				
All transfers	25	(16, 35)	31	(25, 42)
Transfers during labour (before birth)	25	(17, 35)	30	(25, 40)
Potentially urgent transfers (before birth)	24	(15, 30)	30	(24, 40)
Non-urgent transfers (before birth)	27	(20, 36)	35	(25, 45)
Transfers after birth	28	(15, 38)	33	(25, 45)
Potentially urgent transfers (after birth)	30	(20, 44)	30	(20, 40)
**Overall transfer time**^**8 **^**(mins)**				
All transfers	49	(35, 65)	60	(45, 75)
Transfers during labour (before birth)	45	(35, 60)	55	(45, 70)
Potentially urgent transfers (before birth)	42	(30, 55)	50	(40, 65)
Non-urgent transfers (before birth)	50	(37, 65)	60	(50, 75)
Transfers after birth	55	(40, 77)	65	(50, 89)
Potentially urgent transfers (after birth)	54	(40, 70)	60	(45, 75)
**Time to birth after transfer**^**9 **^**(hours)**				
Transfers during labour (before birth)	3.0	(1.2, 7.0)	3.3	(1.4, 7.4)
Potentially urgent transfers (before birth)	1.6	(0.8, 3.0)	1.6	(0.8, 3.1)
Non-urgent transfers (before birth)	5.4	(2.6, 8.8)	6.3	(3.5, 9.3)

^1^Weighted to allow for different durations of data collection.

^2^Time from start of care in labour to decision to transfer.

^3^Fetal distress (1st or 2nd stage or stage not specified), failure to progress in the 2nd stage or antepartum haemorrhage (excluding those where timing of transfer was missing or inconsistent).

^4^Failure to progress in the 1st stage or epidural request (excluding those where timing of transfer was missing or inconsistent).

^5^Time from decision to transfer to start of transfer (when woman left planned place of birth).

^6^Postpartum haemorrhage.

^7^Time from start of transfer to first assessment by midwife or obstetrician in receiving OU.

^8^Time from decision to transfer to first assessment by midwife or obstetrician in receiving OU.

^9^Time from first assessment by midwife or obstetrician in receiving OU to birth (transfers before birth only).

The median overall transfer time, from the decision to transfer to the first OU assessment, was significantly shorter for transfers from home (49 minutes) compared with transfers from FMUs (60 minutes) (p < 0.001). For women transferred before birth, the median time between the woman’s first assessment in the OU and giving birth was around 3 hours in both settings.

### Urgency and transfer duration

**Figure 1:**
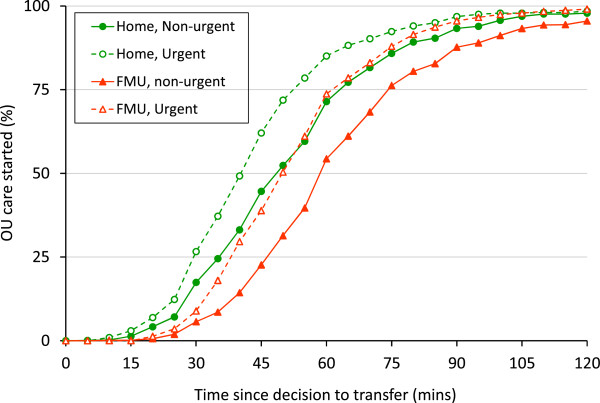
Overall transfer time by urgency in transfers before birth from home and FMUs.

Using our classification of urgency, 668 transfers before birth from home and 642 from FMUs were for potentially urgent reasons; 884 transfers before birth from home and 687 from FMUs were classified as non-urgent. In both settings the overall transfer time was shorter for women transferred before birth for potentially urgent reasons compared with women transferred before birth for non-urgent reasons (home median 42 vs 50 minutes, p < 0.001; FMU median 50 vs 60 minutes, p < 0.001) (Table 3). The shorter transfer times for transfers from home were such that women transferred from home for non-urgent reasons had the same transfer time as women transferred from an FMU for potentially urgent reasons (Table 3 and Figure 1).

For women transferred before birth for potentially urgent reasons the median time from their first assessment in the OU to giving birth was just over 90 minutes in both settings (Table 3).

Transfers after birth for postpartum haemorrhage are also potentially urgent. In these transfers (141 from home and 90 from FMUs) the median overall transfer time was 54 minutes from planned home births and 60 minutes from FMUs (Table 3).

### Urgency and outcomes

In women transferred before birth for potentially urgent reasons from home, 13.4% had an instrumental birth and 2.8% a caesarean within 60 minutes of the start of OU care; similar proportions transferred before birth from FMUs for potentially urgent reasons had an instrumental birth (15.7%) or caesarean (3.0%) within 60 minutes of the start of OU care (Table 4, with further detail including comparable data for AMUs in Additional file 1: Table S1 and Additional file 2: Table S2). Instrumental or caesarean birth within 60 minutes of the start of OU care was much less common in women transferred for non-urgent reasons (Home: instrumental 1.0%, caesarean 0.2%; FMU: instrumental 0.6%, caesarean 0.4%).

**Table 4:** Interventions and outcomes in births within 60 minutes of start of OU care after transfer

		**Instrumental birth**			**Caesarean birth**			**Adverse neonatal outcome**^**1**^			**Apgar <7 at 5 minutes**		
	**Transfers**	**n**	**%**^**2**^	**95% CI**	**n**	**%**^**2**^	**95% CI**	**n**	**%**^**2**^	**95% CI**	**n**	**%**^**2**^	**95% CI**
**Home (N = 16415 births**^**3**^**)**													
Potentially urgent transfers (before birth)	633	82	13.4	10.2-16.6	17	2.8	1.5-4.1	16	2.7	1.0-4.5	9	1.5	0.4-2.6
Non-urgent transfers (before birth)	844	9	1.0	0.3-1.6	1	0.2	0.0-0.5	1	0.1	0.0-0.3	2	0.2	0.0-0.6
All transfers starting before birth	2181	105	4.9	3.7-6.0	36	1.9	1.1-2.7	21	1.2	0.5-1.9	17	1.0	0.4-1.6
**FMU (N = 11085 births**^**3**^**)**													
Potentially urgent transfers (before birth)	616	100	15.7	13.0-18.3	15	3.0	1.2-4.9	11	1.8	0.6-3.0	6	1.0	0.1-1.9
Non-urgent transfers (before birth)	656	5	0.6	0.1-1.1	3	0.4	0.0-0.8	0	0.0		0	0.0	
All transfers starting before birth	1760	118	6.3	5.2-7.5	31	1.8	1.2-2.5	15	0.8	0.4-1.2	6	0.3	0.1-0.6

^1^Intrapartum stillbirth, neonatal admission (within 48 hours of birth) or early neonatal death.

^2^Weighted% of transfers in that category from each setting.

^3^Includes only those women with no inconsistencies in recorded transfer times.

In this group of women who gave birth within 60 minutes of the start of OU care after transfer, adverse neonatal outcomes were uncommon, but a higher proportion of those transferred for potentially urgent reasons had a baby who was admitted to neonatal care or who was stillborn or died in the neonatal period (Home: 2.7%; FMU: 1.8%) compared with women transferred for non-urgent reasons (Home: 0.1%; FMU: 0). Most of these adverse neonatal outcomes (85%) were admissions to a neonatal unit. The number of babies with an Apgar score of less than seven at five minutes in this group was also small, but showed a similar pattern with higher proportions in women transferred for potentially urgent reasons (Home: 1.5%; FMU: 1.0%) compared with women transferred for non-urgent reasons (Home: 0.2%; FMU: 0). Overall, as a proportion of births planned in each setting this represents around 1–2 adverse neonatal outcomes in babies born within 60 minutes of the start of OU care per 1000 low risk births planned at home or in an FMU (Additional file 2: Table S2).

In both groups some women gave birth during transfer. Although numbers were small, in the home birth group this appeared more common in women transferred for potentially urgent reasons (8/633 (1.2%) potentially urgent transfers vs 1/844 (0.1%) non-urgent transfers in the home birth group and 3/616 (0.3%) potentially urgent transfers and 1/656 (0.2%) non-urgent transfers in the FMU group). Two adverse neonatal outcomes occurred in babies born during transfer, both in transfers from home where the reason for transfer did not fall into either the potentially urgent or the non-urgent group.

Transfers for postpartum haemorrhage may also be urgent. Of the women transferred for postpartum haemorrhage, 19.3% of those transferred from home and 19.1% of those transferred from FMUs subsequently received a blood transfusion.

### Distance from the nearest OU and transfer duration from FMUs

Around two thirds of FMUs were located within 20-40 km of the nearest OU in the same NHS trust, with a small number located further than 40 km away. These more distant FMUs accounted for around 2% of planned FMU births.

As might be expected the median time from departure to first OU assessment was well correlated with distance to the nearest OU (r_s_ = 0.62, p < 0.001), while median overall transfer time, which included time arranging the transfer, was more variable (r_s_ = 0.52, p < 0.001).

**Figure 2:**
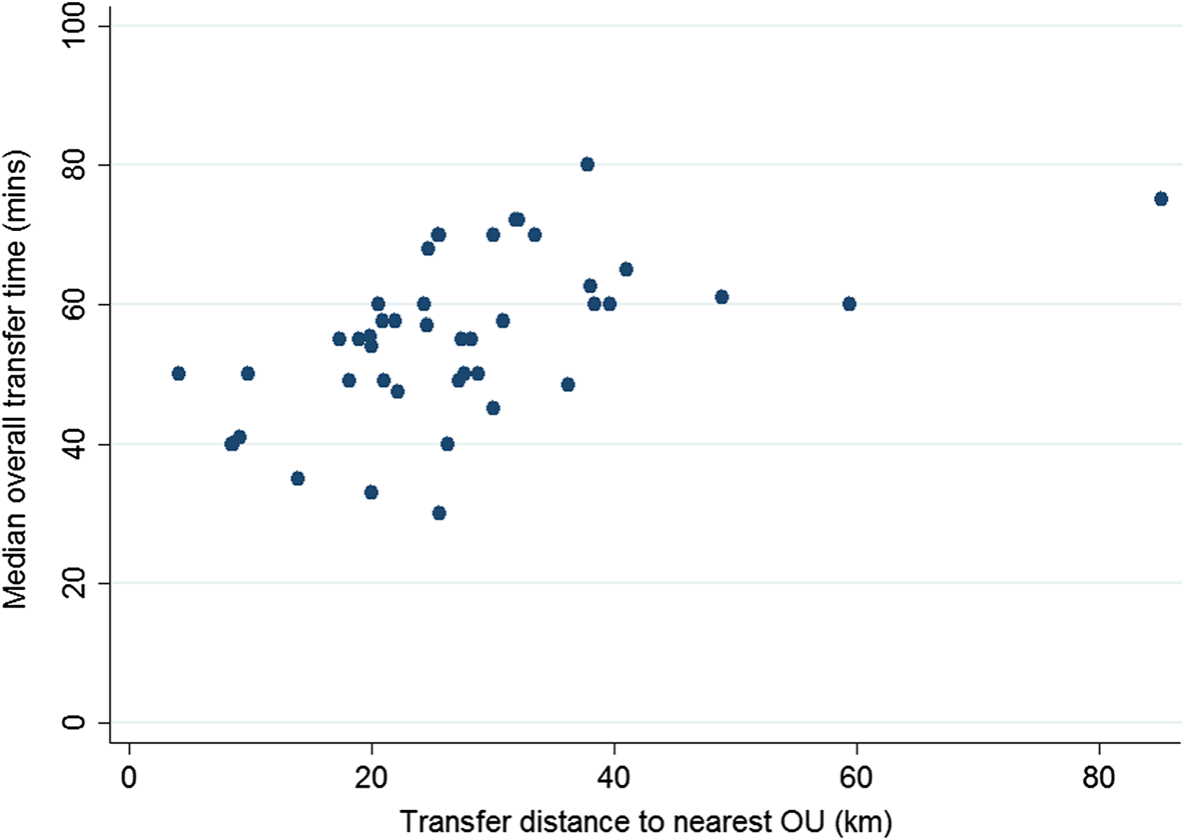
Median overall transfer time for potentially urgent transfers before birth from each FMU, by distance to the nearest OU.

Looking only at transfers before birth for potentially urgent reasons, the median overall transfer time from FMUs located within 20 km of the nearest OU was 47 minutes, increasing to 55 minutes from FMUs situated between 20 and 40 km from the nearest OU and 61 minutes in the small number of FMUs units located more than 40 km away from the nearest OU (Figure 2).

## Discussion

### Main findings

The median overall transfer time, including time spent arranging transfer, waiting for the ambulance to arrive, travel time and any wait before first assessment in the OU, was 60 minutes for transfers from FMUs and 49 minutes for transfers from home. In both settings, the overall transfer time was slightly shorter for transfers before birth for potentially urgent reasons (median 50 minutes from FMUs, 42 minutes from home). Instrumental delivery (forceps or ventouse) within 60 minutes of being transferred occurred in 5-6% of transfers before birth and just under 2% of women transferred before birth gave birth by caesarean section within 60 minutes of being assessed in the OU.

Most FMUs were located within 40 km of the nearest OU and more distant FMUs accounted for a very small proportion of planned FMU births. Distance had some impact on transfer times. The median overall transfer time in transfers for potentially urgent reasons from FMUs located within 20 km of the nearest OU was 8 minutes shorter, at 47 minutes, than for FMUs located between 20 and 40 km away (55 minutes), increasing to 61 minutes in the small number of FMUs located over 40 km away.

### Strengths and limitations

A strength of this study is that it is based on a large sample of planned home and midwifery unit births. Data were collected by attending midwives on a high proportion of eligible women in most participating units and NHS trusts [2], and when women were transferred data collection continued during and after transfer.

One limitation is that only a relatively limited number of data items about transfer were collected. The available data enabled us to evaluate the time taken to arrange transfer in each setting and to evaluate the overall time from decision to transfer to time of first assessment by a midwife or obstetrician in an OU, but because data were not collected on the time of arrival at the OU, we were unable to determine the extent to which delays occurred once the woman had arrived in the OU. In the absence of data on the urgency of transfers we had to operationalise a classification for transfers which used the primary reason for transfer to infer potential urgency or non-urgency. Within the potentially urgent category some transfers will have been more urgent than others, some may have been emergencies, and some transfers for reasons that we did not classify as potentially urgent may also have been urgent. Our analyses on mode of delivery and outcomes in transfers for different reasons lend some support to this classification, but transfers defined by us as potentially urgent should not be considered as emergencies.

For the analysis of the association between distance and transfer duration from FMUs we used the distance to the nearest OU in the same trust as the estimated transfer distance. In a proportion of cases the woman might have transferred to a more distant OU or possibly to a nearer OU in an adjacent trust. The regulatory approvals for the cohort study did not permit collection of women’s postcodes, so we were unable to analyse transfer times by distance for home births.

Data on the timing of the transfer process were recorded by attending midwives. The data were checked for obvious time or date sequence errors; where these could not be corrected the record was excluded from analyses of duration. Some implausibly short and long transfers remained; in some cases likely explanations could be inferred, but some are likely to reflect data recording errors or rounding. Given the methods used, using the median and interquartile range to describe transfer durations, this is not likely to have made a substantial difference to the results.

### Interpretation

The Birthplace study evaluated the safety of planned birth in different settings using an ‘intention to treat’ approach, so the reported comparative risks of adverse perinatal outcomes [1,2] implicitly take account of any risks associated with transfer or with giving birth in a setting without immediate access to obstetric or neonatal services. The transfers described here do not therefore represent any additional risk over and above those already quantified in the Birthplace study which found that for planned births in freestanding midwifery units there were no significant differences in adverse perinatal outcomes compared with planned birth in an obstetric unit; and that for planned home births, adverse perinatal outcomes did not differ for multiparous women, but that for a woman having a first baby, planned home birth significantly increased the risk to the baby. Previous analyses of maternal outcomes in this cohort have also shown that ‘low risk’ women who plan birth at home or in an FMU do not have an increased risk of blood transfusion or admission to higher level care [1,2].

The rates of transfer seen in the Birthplace study, particularly in nulliparous women, are relatively high compared with some other studies, but given the national coverage of the Birthplace cohort study these rates reflect clinical practice in the NHS in England [22-24]. There is no national policy or guidance on what is an acceptable duration for transfer and local NHS guidelines on transfer are of variable quality [25]. Transfer times of 40–50 minutes for potentially urgent reasons may raise concerns that women planning birth in a community setting are exposed to unnecessary risk, so it is important to estimate what proportion of women may need urgent transfer. Data from the Caesarean Section Sentinel Audit suggest that around 1.7% of all births were carried out by caesarean section for a reason which constituted “an immediate threat to the life of the mother or fetus” [26], but this includes women at higher risk of complications who would not be advised to plan birth in an out of hospital setting. Our data on caesareans performed within one hour of the start of care in the OU after transfer suggest that the figure in low risk women is likely to be less than 4 per 1000.

The small proportion of potentially urgent transfers which result in an instrumental or caesarean birth within 60 minutes of the start of care in the OU indicates that transfer for potentially urgent reasons should not be equated with transfer for an obstetric emergency. There is very little evidence on the incidence of obstetric emergencies in low risk births planned at home or in midwifery units although some studies of transfers from midwifery units give some indication of the incidence of complications necessitating urgent transfer. In a study of birth centres in Germany from 1999 to 2001 11.4% of transfers were categorised by midwives as “emergencies” and 10% of babies required neonatal care after transfer.[27] In a cohort study of women planning birth in birth centres in America, 9 per 1000 women who started care in birth centres had an “emergency” transfer during labour and overall, including postpartum transfers for maternal and neonatal reasons, around 2% of women had an “emergency” transfer [28]. The authors noted however that not all these urgent transfers were for indications which could be described as true “medical emergencies”. Mahmood found that only one third of births which took place in the first hour of transfer from an AMU were considered “urgent” by midwives [29].

Concerns have been expressed about long transfer times from distant FMUs [8], but evidence from Scotland where some units are very remote [19] indicates that midwives in more remote units take account of distance and are more cautious in their decision-making about transfer [30] and consider local geography, traffic and weather conditions when making transfer decisions [31]. Nevertheless women are concerned about the duration of transfer, find longer transfer journeys more difficult and may underestimate how long transfers actually take [18].

Given the transfer times described in this study, all members of the multi-disciplinary team caring for women who are transferred have a responsibility to manage any attendant risk appropriately to maximise safety and to consider the woman’s experience. The benefits of good communication and teamwork in cases of transfer were evident in the Birthplace case studies [16]. Communication of urgency has been noted as an important factor in the variability of decision-to-delivery intervals for urgent caesarean sections [26]; appropriate communication of urgency is also likely to be key to the successful transfer of a woman from a planned home or midwifery unit birth and timely assessment and intervention if required on her arrival at the OU. Effective communication at the handover of care is also important from the point of view of women’s experience [18], but requires OU staff to be informed and available.

Our findings show that it typically takes around 15–20 minutes to arrange a potentially urgent transfer, i.e. from decision to transfer to departure from home or the FMU, with transfers from home arranged more quickly on average than those from an FMU. Although it is reassuring that transfers can generally be arranged quickly for potentially urgent transfers from home, the difference between the settings suggest that action may be required to ensure that avoidable delays do not occur when a woman requires urgent transfer from an FMU.

## Conclusions

Transfers from home or FMU commonly take up to 60 minutes from decision to transfer to first assessment in an OU, even for transfers for potentially urgent reasons. However, the possible impact of these transfer times on outcomes is unclear, since the Birthplace primary analysis found similar rates of adverse perinatal outcomes in planned FMU and AMU births, even though urgent transfers can potentially be achieved within minutes in the latter setting.

We do not know if transfer delays contribute to the higher perinatal risks already observed in nulliparous women planning a home birth, but transfers from home are typically achieved more rapidly compared with transfers from FMUs, indicating that in general access to obstetric or neonatal care is not worse for planned home births.

Most transfers from home or FMU are not urgent and emergencies are uncommon, but urgent transfer is more likely for nulliparous women. All women planning birth at home or in an FMU, but particularly women having a first baby, need to be prepared for the possibility of transfer and should be given straightforward information about the potential duration of transfer, including time taken to arrange the transfer and wait for transport.

When women are transferred, effective and timely communication between community, midwifery unit and OU midwives and obstetric colleagues, with particular reference to urgency, is essential to ensure that women receive timely assessment and intervention on arrival at the OU. Development and testing of a standard classification for the urgency of transfer, along the lines of the recommended and widely used classification for the urgency of caesarean section [26], might be one way to facilitate and optimise this communication.

## Competing interests

The authors declare that they have no competing interests.

## Authors’ contributions

This study is part of a programme of work, the research questions and protocol for which was developed by a co-investigator group including JH, RR, PB, MK, AM, CM, MN, MR, JS and LS. JH and RR conceived and developed the outline for this study; JH, RR and JT developed the analysis plan; JT conducted the analysis; RR and JH drafted the manuscript with input from all authors. All authors were involved in interpretation of data, review and revision of the draft manuscript and approval of the final version.

## Pre-publication history

The pre-publication history for this paper can be accessed here:

http://www.biomedcentral.com/1471-2393/13/224/prepub

## Supplementary Material

Additional file 1 **Table showing interventions and outcomes in births within 60 minutes of start of OU care after transfer, as a proportion of transfers from home, FMUs and AMUs.** This table shows the data provided in Table 4 alongside comparable data on transfers from AMU. Click here for file

Additional file 2 **Table showing interventions and outcomes in births within 60 minutes of start of OU care after transfer, as a proportion of all births planned at home, in FMUs and AMUs.** The same data as in additional file 1: Table S1, but as a proportion of all births planned in each setting. Click here for file
